# Identification, Bioinformatics, and Expression Analysis of *JAZ* Gene Family in Flax (*Linum usitatissimum* L.)

**DOI:** 10.3390/ijms27083594

**Published:** 2026-04-17

**Authors:** Liuxi Yi, Ying Sun, Yu Zhou, Yingnan Mu, Wenyu Han, Yuheng Dong, Huiqing Lan, Jianping Zhang, Yongsheng Chen

**Affiliations:** 1 Agricultural College, Inner Mongolia Agricultural University, Hohhot 010019, China; yiliuxivip@163.com (L.Y.);; 2 Inner Mongolia Academy of Agricultural & Animal Husbandry Sciences, Hohhot 010031, China; 3 Tongliao Academy of Agricultural and Animal Husbandry Sciences, Tongliao 028015, China; 4 Crop Research Institute, Gansu Academy of Agricultural Sciences, Lanzhou 730070, China

**Keywords:** flax, JAZs, whole genome conserved domains, bioinformatics, transcriptome analysis

## Abstract

Jasmonate ZIM-domain (JAZ) proteins, as core negative regulatory factors of the jasmonic acid (JA) signaling pathway, play a key role in the growth and development of plants and the response to biotic and abiotic stress. In this study, 11 flax JAZ members were identified, all of which contain a ZIM domain and a Jas domain. LuJAZs comprise 3–16 exons, encoding 187–808 amino acids (aa) with molecular weights ranging from 20.24 to 88.76 kDa and isoelectric points (PI) of 5.68–9.77. They are all hydrophilic proteins located in the nucleus. These 11 LuJAZ genes are divided into five subfamilies and are unevenly distributed on chromosomes. Transcriptome and qRT-PCR analyses revealed that six *LuJAZ* genes, including *LUSG00004384*, *LUSG00030782*, *LUSG00016742*, *LUSG00004390*, *LUSG00010997*, and *LUSG00029783*, are significantly induced by JA. The protein–protein interaction (PPI) prediction and analysis of differential expression genes (DEGs) suggest that the *MYC2* gene (*LUSG00028070*) may play a role in the JA-induced response. This study provides a theoretical basis for further exploring the function of the JAZ family in flax.

## 1. Introduction

Flax (*Linum usitatissimum* L.) is an annual herbaceous crop belonging to the family Linaceae. Flax stems can produce high-quality fibers with a strength two to three times that of cotton fibers, playing an important role in global agriculture and textile industries [1,2]. Linseed contains about 30–40% oil, 20–25% protein, and 20–28% fiber and minerals [3]. Linseed oil is rich in linolenic acid, omega-3 fatty acids, omega-6 fatty acids, and vitamins A, B, D, and E, and has a high nutritional value [3]. Linseed is considered to help digestion and reduce the risk of cardiovascular diseases, especially type 2 diabetes [4]. Linseed “milk” is emerging on the market. It is suitable for people who are intolerant to gluten, nuts, or soybeans, and is healthier than almond milk [4,5,6,7,8]. Due to its potential health benefits, linseed has received increasing attention in the past 20 years [9].

Many abiotic and biotic stresses that are persistent threats to plants affect the quality and yield of flax. Jasmonic acid (JA) plays multiple roles in plant growth, fertility, and environmental adaptability, and its content increases after being attacked by herbivores, pathogens, and mechanical damage [10,11,12,13,14,15,16]. The increase in JA content is usually accompanied by upregulation of defense-related gene expression, ultimately inducing many secondary metabolites with defense functions, such as chitosan, terpenoid indole alkaloids, nicotine, indole glucoside, flavonoids, and anthocyanins [16,17,18,19].

**Figure 1:**
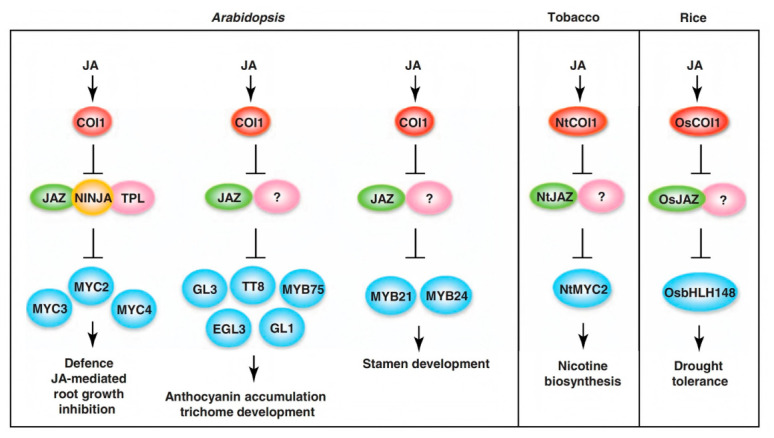
JAZ repressors through interaction with diverse transcriptional activators and co-repressors modulate diverse jasmonate-dependent functions [36]. Note: Coronatine Insensitive 1; GL1, GLABRA1; GL3, GLABRA3; EGL3, enhancer of GLABRA3; MYC2, MYC3 and MYC4, basic helix–loop–helix (bHLH) transcription factors; NINJA, novel interactor of JAZ; JA, jasmonate; JAZ, jasmonate-ZIM domain; TT8, transparent test A8. Arrows indicate positive regulation; T-bars represent negative regulation; The question marks denote unknown regulatory relationships.

JAZ protein belongs to the plant TIFY protein family and is a key negative regulator of JA signaling. The JAZ protein family includes ZIM domains and Jas domains [20]. The ZIM domain consists of 30 amino acids in the central part of the JAZ peptide sequence and contains a highly conserved TIFY motif [21]. The TIFY motif has been confirmed to be essential for the inhibitory activity of various JAZ proteins and for the formation of homodimers and heterodimers within the JAZ protein family [22,23]. The Jas domain is highly conserved in the JAZ protein family and is involved in most protein–protein interactions [24]. In the JA pathway, JAZ protein plays a central role in the cascade reaction between upstream JA signaling and downstream transcription factors (TFs) and defense-related genes, forming a JA-dependent signaling pathway (Figure 1) [25,26]. Upon perceiving bioactive JAs, the JA receptor Coronatine Insensitive 1 (COI1) targets JAZs for destruction, releasing downstream TFs to regulate transcription and JA responses [27].

The JAZ protein family is widely present in plants such as Arabidopsis [28]; rice (*Oryza sativa* L.) [29]; maize (*Zea mays* L.) [30]; potato (*Solanum tuberosum* L.) [31]; tomato (*Solanum lycopersicum*) [32]; etc. At present, research on the function of JAZs in Arabidopsis is relatively thorough. Studies have found that Arabidopsis jaz4-1, jaz7-1, and jaz8-1 mutants accelerate dark leaf-induced leaf aging, while jaz6-1 and jaz6-2 delay leaf aging [33,34]. Jaz4-1 mutants can delay flowering and increase sensitivity to Pst DC3000 [35]. The jaz6-18 and jaz8-10 mutants enhance JA regulated root growth inhibition and defense against the necrotic fungus Botrytis cinerea [28]. However, to date, there have been few reports on the JAZ gene family in flax. Based on a subgenome database, this study screened the LuJAZ family in flax and systematically analyzed their physicochemical properties, gene structure, evolutionary relationships, and expression patterns. The aim was to further investigate the JAZ genes that play a key regulatory role in the JA pathway in flax.

## 2. Results

### 2.1. Identification of Members of JAZ Protein Family in Flax

To identify all the JAZ family proteins in flax, the Pfam database was used to find the seed file of JIM (PF0200) and jas (PF09425) structural domains. Finally, a total of 11 JAZ family members were identified from the flax genome (Table 1). Physicochemical property analysis showed that the amino acid number of LuJAZ proteins ranged from 187~808 aa, with LUSG000030782 being the shortest (187 aa) and LUSG00029029 being the longest (808 aa). The weight ranges from 20.24 to 88.76 kDa. The isoelectric point (pI) was between 5.68 (LUG00004390) and 9.77 (LUSG00010997). Among the 11 LuJAZ proteins, 1 member was acidic (pI < 7) and 10 members were alkaline (pI > 7). The average hydrophobicity (GRAVY) of LuJAZs was negative (−0.786~−0.360), indicating that they were all hydrophilic proteins. Subcellular localization prediction showed that 11 LuJAZs were all in the nucleus (Table 1), and it can be speculated that LuJAZ proteins played a transcriptional regulatory function through nuclear localization.

### 2.2. Gene Structure and Conserved Domain Analysis

**Figure 2:**
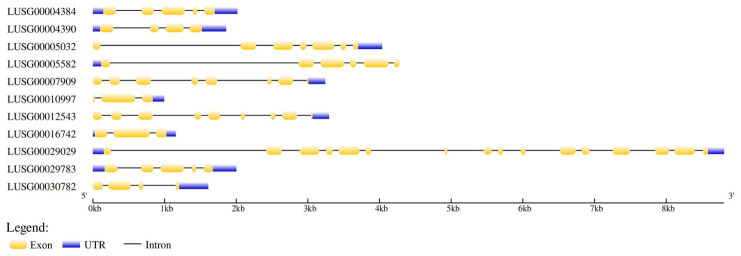
The genetic structure of *LuJAZs*.

The analysis of the structure of 11 *LuJAZ* genes using GSDS 2.0 showed that the number of exons of *LuJAZ* genes varied significantly, ranging from 3 to 16, with an average of 6 exons. Among them, *LUSG00010997* and *LUSG00016742* contained the least number of exons, with 3 exons and 2 intron structures; conversely, *LUSG00029029* contained 16 exons and 15 introns, with the most exons among the members (Figure 2). These results suggest that *LuJAZ* genes have undergone significant structural differentiation during evolution.

**Figure 3:**
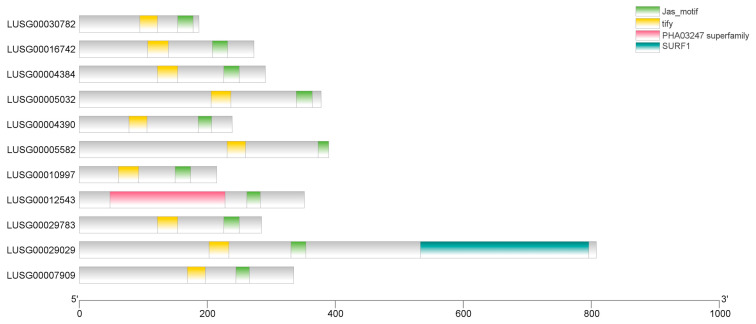
The domain analysis of LuJAZs.

**Figure 4:**
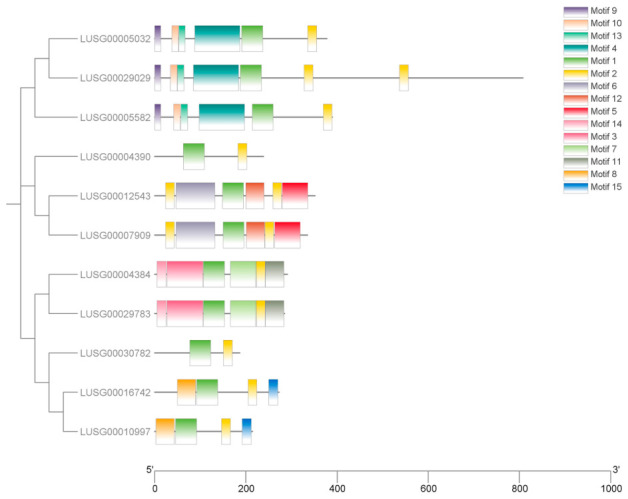
The motif analysis of LuJAZs.

Conserved domain and motif analysis revealed that all LuJAZ members contained a typical ZIM domain (composed of about 25 amino acids with “C2C2” zinc finger motif) and a Jas domain (located at the C-terminus, about 28 amino acids with a core sequence of “SL2FX2KRX2RX5PY”) (Figure 3 and Figure S1). MEME analysis identified 15 conserved motifs (Motif 1~15) in LuJAZs, of which Motif 1 corresponded to the ZIM structural domain, Motif 2 corresponded to the Jas structural domain, and Motif 3~15 were family-specific motif. All members contained Motif 1 and Motif 2 (Figure 4), which further confirmed the conservation of the JAZ family.

### 2.3. Analysis of Cis-Elements

**Figure 5:**
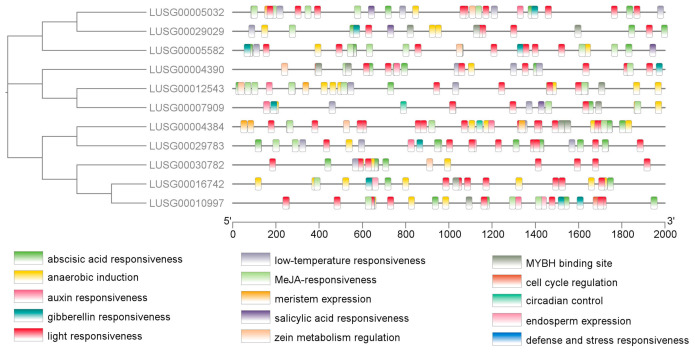
Analysis of the cis-elements of *LuJAZs*.

The PlantCARE website was used to analyze the cis-acting elements of the upstream 2000 bp promoter region of the LuJAZ genes. A total of 15 kinds of cis-acting elements were identified on 11 LuJAZ genes (Figure 5). They included abscisic acid response; anoxia induction; auxin response; gibberellin response; light response; low-temperature response; jasmon methylester response; meristem expression; salicylic acid response; zein; MYBH binding site; cell cycle regulation; circadian regulation; endosperm expression; and defense and stress response-related elements.

### 2.4. Phylogenetic Analysis of LuJAZs

**Figure 6:**
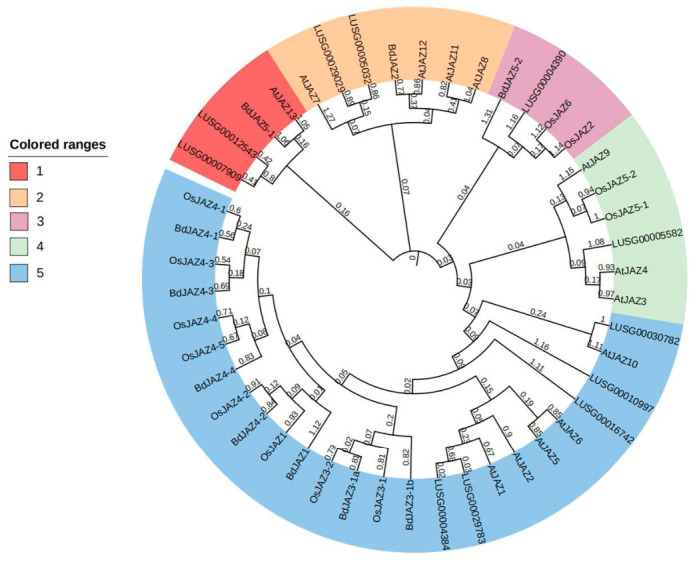
The phylogenetic analysis of the JAZ protein family in flax, Arabidopsis, rice, and *Brachypodium distachyon*.

To study the systematic relationship of LuP450, the LuJAZ protein sequences were separately constructed with 12 Arabidopsis JAZ, 12 rice OsJAZ, 11 *Brachypodium* BdJAZ protein sequences to construct a phylogenetic tree (Figure 6). The results showed that all JAZ proteins could be divided into five subfamilies (Subgroup 1~5). Subgroup 5 had the most members (25), whereas Subgroups 1 and 2 had the fewest (4 each).

LuJAZ proteins were unevenly distributed among the subgroups: Subgroup 1 contained 2 members (LUSG00012543 and LUSG00007909); Subgroup 2 contained 2 members (LUSG00029029 LUSG00005032); Subgroup 3 contained 1 member (LUSG00004390); Sub 4 contained 1 member (LUSG00005582); and Subgroup 5 contained 5 members (LUSG00030782, LUSG00010997, LUSG00016742, LUSG00029783, LUSG00004384).

### 2.5. Chromosomal Localization and Gene Duplication Analysis

**Figure 7:**
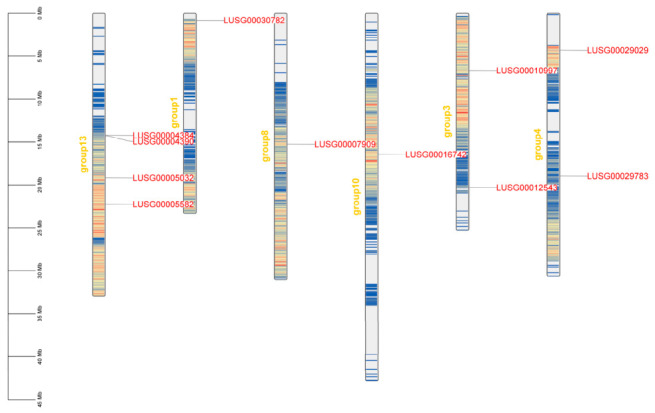
Chromosomal localization of the *LuJAZ* genes. Note: group13 represents Chr13; group1 represents Chr1; group8 represents Chr8; group10 represents Chr10; group3 represents Chr3; and group4 represents Chr4.

Chromosome localization analysis showed that the 11 *LuJAZ* genes were unevenly distributed in the six chromosomes of flax. Chr13 contained four genes (*LUSG00004384*, *LUSG00004390*, *LUSG00005032*, and *LUSG00005582*), which was the most densely distributed chromosome of *LuJAZs*. Chr3 contained two genes, *LUSG00010997* and *LUSG00012543*. Chr4 also contained two genes, *LUSG00029029* and *LUSG00029783*. Chr1, Chr8, and Chr10 each contained one gene of *LUSG00030782*, *LUSG00007909*, and *LUSG00016742*, respectively (Figure 7). In addition, a significant gene cluster was present in the chromosome: *LUSG00004384* and *LUSG00004390* on Chr13 (Figure 7).

### 2.6. The Transcriptome Analysis

To further study the response of *JAZs* to JA, this study utilized transcriptome sequencing to determine the expression of *JAZs* after MeJA treatment for 0 h, 12 h, and 24 h (9 samples). The results showed that an average of 737 Gb of raw_bases were generated per sample, of which 7.18 Gb were clean reads, and the average alignment rate of clean reads was 97.97%. The Q20 and Q30 of effective reads were 99.45% and 97.50%, respectively (Table S1). The clean reads had an alignment rate of 97.12% with the reference genome, indicating that most of the transcripts were completely covered (Table S1).

To analyze the differences between samples, the Pearson correlation coefficient of all gene expressions between each two samples was calculated, and the correlation between replicates of samples treated with MeJA for 0 h, 12 h and 24 h was higher than 90.5%, 96.4%, and 90.2%, respectively, indicating good reproducibility between the samples (Figure S2).

**Figure 8:**
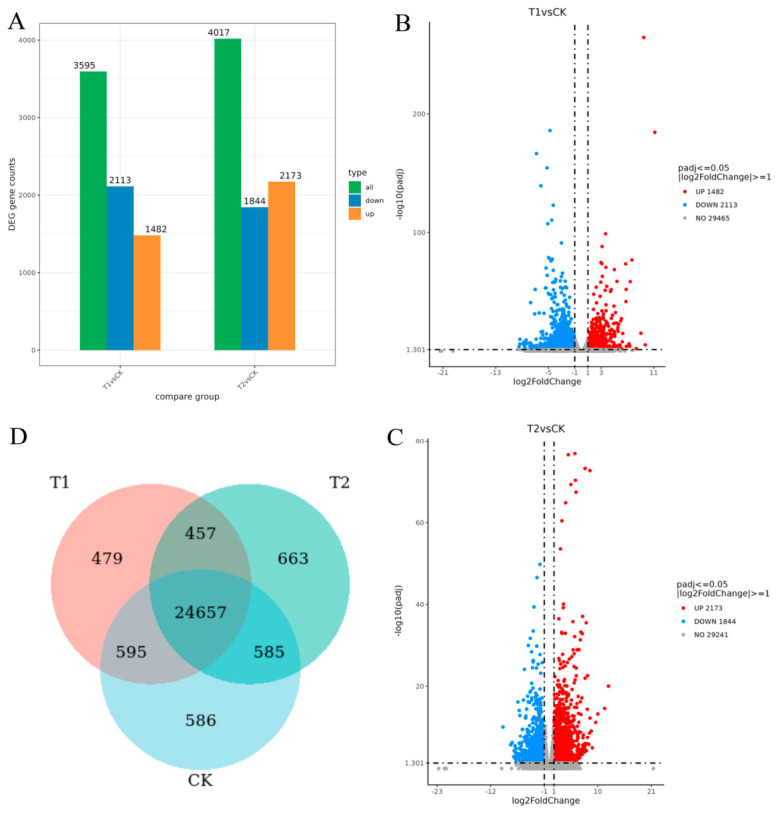
DEGs after MeJA treatment for 0 h, 12 h, and 24 h. Note: DEGs after MeJA treatment for 0 h, 12 h, and 24 h. (**A**) The bar plot of the number of DEGs in the comparison group; (**B**) the volcano plot of DEGs after MeJA treatment for 0 h and 12 h; (**C**) the volcano plot of DEGs after MeJA treatment for 0 h and 24 h; (**D**) the Venn diagram of after MeJA treatment between 0 h and 12 h and between 0 h and 24 h.

Using the DESeq2 method, DEGs at different time points after treatment with MeJA were analyzed. After screening by padj ≤ 0.05, it was found that there were 3595 DEGs between 0 h and 12 h, of which 2113 genes were up-regulated and 1482 genes were down-regulated. There were 4017 DEGs between 0 h and 24 h, of which 1844 genes were up-regulated and 2173 genes were downregulated (Figure 8). Through the cluster analysis of DEGs, it can be observed that a large number of genes have significant differences in expression at 0 h, 12 h, and 24 h.

**Figure 9:**
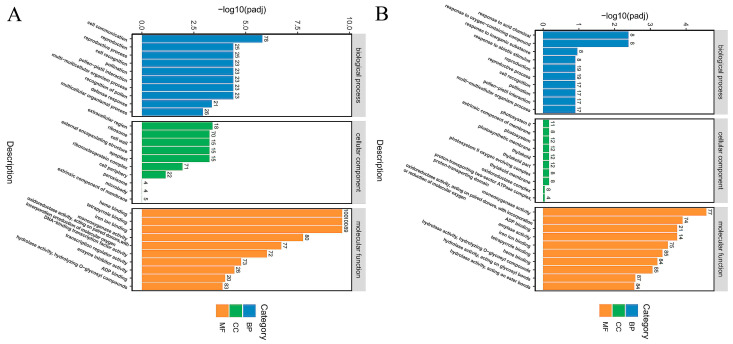
GO enrichment analysis bar plot of DEGs. Note: 12 h and 24 h of MeJA treatment. (**A**) GO enrichment analysis bar plot of DEGs between 0 h and 12 h; (**B**) GO enrichment analysis bar plot of DEGs between 0 h and 24 h.

DEGs were mapped to each item of the Gene Ontology database (http://www.geneontology.org/, accessed on 22 October 2025). The most significant 30 GO items were selected for analysis after screening with the padj ≤ 0.05 threshold. The GO function enrichment classification showed the DEGs were mainly involved in three categories: biological process, cellular component, and molecular function (Figure 9). Among them, the DEGs involved in biological processes were analyzed, and the results showed that the DEGs between 0 h and 12 h were significantly enriched in 10 types of biological processes, mainly including cell communication, reproduction, reproductive process, cell recognition, pollination, pollen-pistil interaction, multi-multicellular organism process, recognition of pollen, defense response and multicellular organismal process (Figure 9A). The DEGs between 0 h and 24 h were significantly enriched in 10 types of biological processes, including the response to acid chemical; response to oxygen-containing compound; response to inorganic substance; response to abiotic stimulus; reproduction; reproductive process; cell recognition; pollination; pollen–pistil interaction; and multi-multicellular organism process. GO enrichment analysis of DEGs between 0 h and 12 h as well as 0 h and 24 h showed MeJA treatment affected the reproduction, reproductive process, cell recognition, pollination, pollen-pistil interaction, and multi-multicellular organism process (Figure 9B)—at 12 h of MeJA treatment, the defensive response genes were enriched. At 24 h, the genes response to acid chemical, response to oxygen-containing compound, response inorganic substance, and response to abiotic stimulus were enriched. These results indicate that MeJA treatment activated the expression of defense response and various endogenous and exogenous stimulus genes.

**Figure 10:**
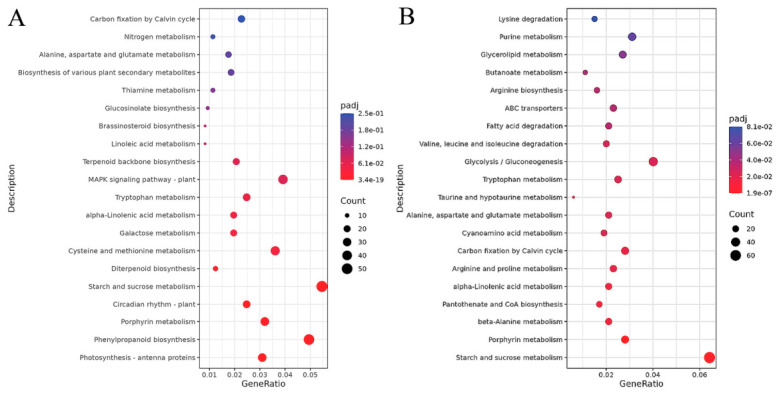
KEGG enrichment analysis of DEGs. Note: (**A**) KEGG enrichment pathways of DEGs after 12 h of MeJA treatment; (**B**) KEGG enrichment pathways of DEGs after 24 h of MeJA treatment. The *x*-axis represents the ratio of the number of DEGs annotated to the KEGG pathway to the total number of DEGs. The *y*-axis represents the KEGG pathway.

DEGs were mapped to the various metabolic pathways of the KEGG database. With the threshold of padj ≤ 0.05, the KEGG pathway enrichment analysis of DEGs was performed. The results showed that at 12 h of MeJA treatment, DEGs were mainly enriched in carbon fixation by Calvin cycle; nitrogen metabolism; alanine, aspartate, and glutamate metabolism; biosynthesis of various plant secondary metabolites; thiamine metabolism; glucosinolate biosynthesis; brassinosteroid biosynthesis; linoleic acid metabolism; terpenoid backbone biosynthesis; MAPK signaling pathway–plant; tryptophan metabolism; alpha-linolenic acid metabolism; galactose metabolism; cysteine and methionine metabolism; diterpenod biosynthesis; starch and sucrose metabolism; circadian rhythm–plant, porphyrin metabolism, phenylpropanoid biosynthesis; and photosynthesis–antenna proteins (Figure 10A). At 24 h of MeJA treatment, DEGs were mainly enriched in lysine degradation; purine metabolism; glycerolipid metabolism; butanoate metabolism; arginine biosynthesis; ABC transporters; fatty acid degradation; valine, leucine, and isoleucine degradation; glycolysis/gluconeogenesis; tryptophan metabolism; taurine and hypotaurine metabolism; alanine, aspartate, and glutamate metabolism; cyanoamino acid metabolism; carbon fixation by Calvin cycle; arginine and proline metabolism; alpha-linolenic acid metabolism; pantothenate and CoA biosynthesis; beta-alanine metabolism; porphyrin metabolism; and starch and sucrose metabolism. (Figure 10B). The results showed that after 12 h and 24 h of MeJA treatment, DEGs were significantly enriched in the alpha-linolenic acid metabolism (ko00592) and starch and sucrose metabolism (ko00500) pathways, indicating that MeJA treatment not only affects JA synthesis but also influences plant carbon metabolism.

Furthermore, the analysis of the flax LuJAZ genes showed that six genes, including *LUSG00004384*, *LUSG00030782*, *LUSG00016742*, *LUSG00004390*, *LUSG00010997* and *LUSG00029783*, were significantly up-regulated after treatment with MeJA for 12 h and 24 h. *LUSG00005032* was significantly up-regulated after treatment with MeJA for 12 h, but it did not reach a significant level at 24 h (Table S2). *LUSG00005582*, *LUSG00012543*, *LUSG00029029*, and *LUSG00007909* did not significantly change after treatment with MeJA for 12 h and 24 h (Table S2). This indicated that the six genes, *LUSG00004384*, *LUSG00030782*, *LUSG00016742*, *LUSG00004390*, *LUSG00010997*, and *LUSG00029783*, might play an important role in response to the JA pathway.

### 2.7. qRT-PCR

**Figure 11:**
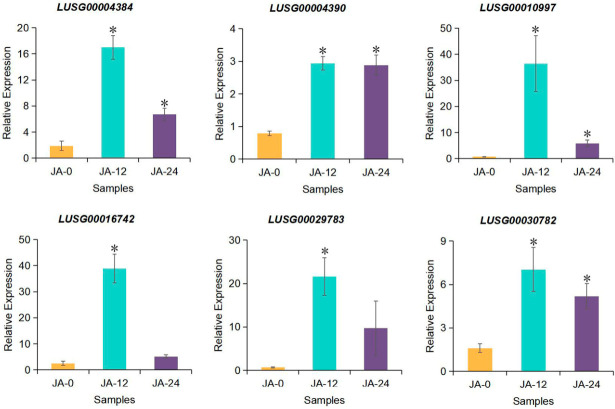
The qRT-PCR verification of the expression of *LuJAZs.* Asterisks indicate significant differences between treatment and control (Student’s *t*-test: * *p* < 0.05).

Six key genes of LuJAZs from the transcriptome that responded to JA were further selected. qRT-PCR analysis was performed with LuActin as an internal control (Figure 11). The results showed that *LUSG00016742* and *LUSG00029783* genes were inconsistent with the transcriptome at 24 h. *LUSG00016742* and *LUSG00029783* were consistent with the transcriptome at 12 h and the four genes *LUSG00004384*, *LUSG00030782*, *LUSG00004390* and *LUSG00010997* were consistent with the transcript at 12 h and 24 h. The consistency reached 83.3%, indicating that the expression trend of qRT-PCR was basically consistent with transcriptome, suggesting that the transcriptome data had high reliability (Figure 11). Further, it showed that *LUSG00004384*, *LUSG00030782*, *LUSG00016742*, *LUSG00004390*, *LUSG00010997*, and *LUSG00029783* might play an important role in response to the JA pathway.

### 2.8. The Protein–Protein Interaction Network Based on STRING

**Figure 12:**
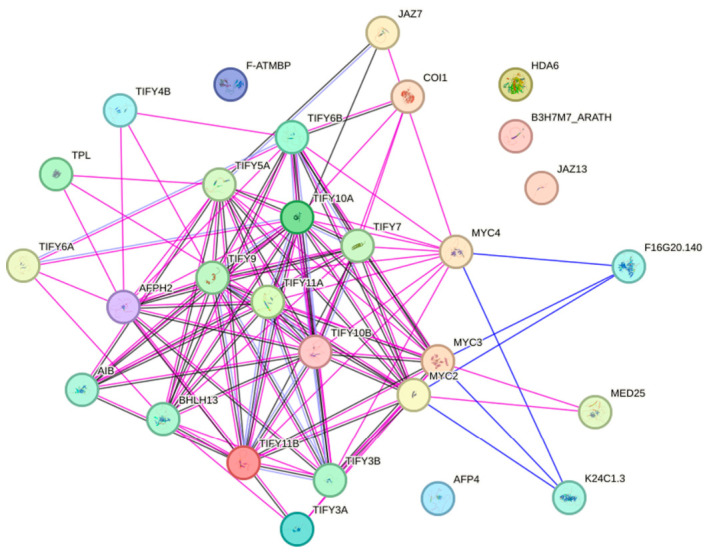
Prediction of the protein–protein interaction network of the JAZ (TIFY) family in flax. Note: Nodes in different colors represent proteins of different functional categories, and lines represent interactions between proteins. The confidence level for interactions is set to medium confidence, which is 0.40.

Based on the STRING database, a protein–protein interaction (PPI) network of the JAZ family proteins in flax was constructed (Figure 12). The results showed that members of the JAZ family in flax (TIFY family proteins) form a tight and complex interaction regulatory network with various functional proteins. Over 20 highly confident interactions were screened. Among them, TIFY6B, TIFY10A, TIFY11B, TIFY5A, TIFY3B, TIFY9 and TIFY10B are core hub proteins in the network, with the highest connectivity and the largest number of interacting proteins, serving as key hub members of the JAZ family interaction network in flax. From the functional classification of interacting proteins, the JAZ family proteins in flax mainly specifically interact with core proteins of the JA signaling pathway, including JA receptor protein COI1, key transcription factors MYC2/MYC3/MYC4, co-suppressors TPL, AIB, AFPH2, epigenetic regulator HDA6, mediator MED25, and BHLH13, as well as other transcription regulatory proteins. This interaction network provides an important theoretical basis for subsequent functional verification of key JAZ genes and analysis of regulatory pathways.

Furthermore, the interacting proteins (predicted by String) of JAZs in DEGs were screened. After 12 h of MeJA treatment, two MYC2 genes were upregulated, namely LUSG00028070 and LUSG00000501. After 24 h of MeJA treatment, two MYC2 genes were upregulated, namely LUSG00033635 and LUSG00028070 (Table S3).

## 3. Discussion

Flax has garnered increasing attention due to its significant value in agriculture, textiles, and healthcare [1,2,9]. JA plays a crucial role in regulating plant growth, development, and defense-related processes [27]. The JAZ repressor protein holds a central position in the JA signaling cascade [37]. Currently, the JAZ protein family has been identified in dicotyledonous plants such as Arabidopsis [38,39], cotton [40], and tomato [41], as well as monocotyledonous plants like rice [42] and maize [30]; however, the JAZ family in flax has not been reported. In this study, we screened seed files for the PF06200 ZIM domain and PF0942 jas domain through whole genome conserved domain analysis, identifying a total of 11 LuJAZ proteins in flax. All of them are hydrophilic proteins and localized in the nucleus. Analysis of upstream cis-elements revealed that LuJAZ genes play important roles in tissue-specific expression, hormone response, and stress response. Previous studies have found that JAZ proteins contain a nuclear localization signal (PY-NLS) [43,44]. The LuJAZs we identified in our study fully conform to the characteristics of JAZ proteins, providing a foundation for subsequent research.

The core function of JAZ proteins is to participate in the negative regulation of JA signaling pathway. The functions of JAZ family proteins have been relatively well-studied in Arabidopsis. *AtJAZ11/12* is involved in coordinating the balance between “growth and development” and “defense response”, as well as the development of floral organs [27]. *AtJAZ3* regulates root growth and development through JA signaling homeostasis [27,45]. *AtJAZ5* participates in JA-mediated biological stress response and simultaneously regulates seedling growth [46]. *AtJAZ2* is mainly involved in the regulation of abiotic stress (drought/cold) [47]. *LUSG00012543* and *LUSG00007909* genes cluster with *AtJAZ5*; *LUSG00029029* and *LUSG00005032* cluster with *AtJAZ11/12*; *LUSG00005582* clusters with *AtJAZ3*; and *LUSG00004390* clusters with *AtJAZ2*. Whether the *LuJAZs* in flax have functional similarities with their Arabidopsis thaliana homologs remains to be further studied in the future.

In the JA signaling pathway, JAZ forms a cascade reaction with downstream transcription factors and defense-related genes [25,26]. The endogenous bioactive jasmonoyl-isoleucine (JA-Ile) conjugate mediates the binding of JAZ proteins to the F-box protein Coronatine Insensitive 1 (COI1). Subsequently, the 26S proteasome degrades JAZ proteins, releasing various transcription factors that are inhibited by JAZ and activating their respective downstream responses [36,48]. JAZ interacts with MYC2, MYC3, MYC4, NtMYC2, OsCOI1, and OsbHLH148 to regulate plant defense responses [36,49,50]. JAZ interacts with MYB21, MYB24, PAP1, GL1, GL3, EGL3, and TT8 to regulate plant development [36]. This study found that the *MYC2* gene *LUSG00028070* was upregulated at both 12 h and 24 h after MeJA treatment. Whether LUSG00028070 interacts with LuJAZs and how it affects downstream pathways remains to be further investigated, providing ideas for subsequent research.

## 4. Materials and Methods

### 4.1. Identification of the Members of the Flax JAZ Protein Family

Based on the flax protein family sequence information (https://phytozome-next.jgi.doe.gov/, accessed on 11 September 2025), the ZIM structural domain (PF06200) and Jas structural domain (PF09425) were verified using the Pfam (http://pfam.xfam.org/, accessed on 11 September 2025) online tool. Sequences without core structural domains were excluded. The family members of flax JAZs were finally determined.

### 4.2. Physicochemical Properties and Subcellular Localization Prediction

The amino acid number, molecular weight, isoelectric point, hydrophobicity, and other physicochemical properties of *LuJAZs* were analyzed by ExASy ProtParam tool (https://web.expasy.org/protparam/, accessed on 23 September 2025). The online tool Cell-PLoc 2.0 (http://www.csbio.sjtu.edu.cn/bioinf/Cell-PLoc-2/, accessed on 7 October 2025) was used to predict their subcellular location.

### 4.3. Gene Structure and Conserved Domain Analysis

Based on the CDS sequences of *LuJAZ* genes and the genomic DNA sequences, the gene structure schematic diagram (exon–intron distribution) was drawn using the GSDS2.0 tool (https://gsds.gao-lab.org/, accessed on 12 October 2025). MEME Suite 5.5.3 (https://meme-suite.org/meme/, accessed on 13 October 2025) was used to analyze conserved motifs, with 15 motifs and the default values for the other parameters. The TBtools-II (toolbox for biologists) v2.356 software was used to draw a diagram of the distribution of conserved structural domains and motifs. The cis-acting elements of the LuJAZs were conducted by PlantCARE (http://bioinformatics.psb.ugent.be/webtools/plantcare/html/, accessed on 13 October 2025).

### 4.4. System Evolution Tree Construction

The protein sequences of JAZs of Arabidopsis (At), rice (Os), and barley (Bd) were downloaded [30], and a multiple sequence alignment was performed with flax LuJAZ protein sequences by Clustal 7.0.2.1) (default parameters). The MEGA 11 software was used to construct a phylogenetic tree using the Neighbor-Joining (NJ) method, with a bootstrap value of 100 replicates and other parameters set to default.

### 4.5. Chromosomal Localization and Gene Duplication Analysis

Chromosome positions of *LuJAZs* genes were extracted from the flax genome annotation file, and TBtools software were used to draw the chromosome localization map. Gene duplication events were analyzed using the built-in tool MCScanX (v1.0.0, Bioconda) of tbtools with the E-value set to ≤1 × 10^−10^. Sequences with homology ≥80% were determined to be paralogous genes.

#### 4.5.1. MeJA-Induced Gene Expression Analysis

The flax variety “Neiya 9” selected by our research group was cultivated to the four-leaf stage, and then treated with 50 μM methyl jasmonate (MeJA 392707, Sigma-Aldrich, Merck KGaA, Darmstadt, Germany) by foliar spraying [51]. Samples sprayed with equal amounts of ddH_2_O were used as a control. Samples were collected at 0 h, 12 h, and 24 h, and the leaves of three different plants were used as three biological replicates at each time point. Total RNA was extracted by Trizol method. RNA quality control is performed by Agilent 2100 bioanalyzer (Agilent, Santa Clara, CA, USA). The transcriptome sequencing was performed using Illumina high throughput sequencer (illumina NovaseqXplus) (Illumina, San Diego, CA, USA). The mRNA was enriched with Oligo (dT) magnetic beads and then cut into short fragments (raw reads). Adapter sequences, low-quality reads, and reads with >5% ambiguous bases (N) were removed, resulting in quality reads. The quality reads were spliced and aligned with the flax reference genome (https://phytozome-next.jgi.doe.gov/info/Lusitatissimum_v1_0, accessed on 3 September 2025). HISAT2 (2.2.1) was used to align paired-end reads to the reference genome. The multimapped reads were excluded. FeatureCounts (2.0.6) was used to count ds mapped to each gene. The analysis parameters for HISAT2 (2.2.1) and FeatureCounts (2.0.6) were set to default vales. The FPKM (kilobase fragment count per million mapped reads per transcript) value is calculated and used to estimate the impact of sequencing depth and gene length on read cous. DESeq2R (1.42.0) was used for differential expression analysis. *p*-values were adjusted using the Benjamini and Hochberg method to control the false discovery rate. The threshold for significant differential expression was set at padj ≤ 0.05 and |log2(foldchange)| ≥ 1. GO (Gene Ontology) and KEGG (Kyoto Encyclopedia of Genes and Genomes) enrichment analysis was performed using the ClusterProfiler R package (v4.18.0, Bioconductor). Differential gene set enrichment used predefined gene sets (from functional annotations or results of previous experiments). GSEA (https://www.gsea-msigdb.org/gsea/index.jsp, accessed on 13 November 2026) was used for GSEA analysis of GO and KEGG datasets.

#### 4.5.2. RT-qPCR

The expression level of *LuJAZ* genes was detected by qRT-PCR with *LuActin* as the internal reference gene, and the primer sequences are shown in Table S4. Reaction system: 2× SYBR Green Mix 10 μL, upstream primers 0.5 μL, downstream primers 0.5 μL, cDNA 2 μL, and ddH_2_O 7 μL. Reaction program: 95 °C pre-denaturation for 30 s; 95 denaturation for 5 s; and 60 °C annealing for 30 s, over 40 cycles. Relative expression was calculated by 2^−ΔΔCt^ method, and the expression curve was drawn by GraphPad Prism 9.

## 5. Conclusions

This study identified and characterized the *LuJAZ* gene family in flax. JAZs are core negative regulators of the JA signaling pathway, playing a crucial role in plant stress response and growth and development. However, research on the flax *JAZ* gene family remains scarce. Based on the flax genome, this study identified 11 *LuJAZ* genes, all of which encode hydrophilic nuclear-localized proteins. Significant differentiation exists in the gene structures of family members. Phylogenetic analysis revealed the evolutionary relationships and functional conservation of the *LuJAZ* family. Combined with transcriptome analysis and qRT-PCR validation, it was found that six LuJAZ genes (*LUSG00029783*, *LUSG00016742*, *LUSG00004384*, *LUSG00010997*, *LUSG00004390*, *LUSG00030782*) may be involved in the response of the JA signaling pathway. A predicted MYC2 protein, LUSG00028070, that may interact with LuJAZs was identified.

## Figures and Tables

**Table 1:** The physicochemical properties of LuJAZ family members.

Sequence ID	Number of Amino Acid	Molecular Weight	Theoretical pI	Instability Index	Aliphatic Index	Grand Average of Hydropathicity	Predicted Location(s)
LUSG00030782	187	20,244.87	9.02	60.99	80.32	−0.397	Nucleus
LUSG00016742	273	28,700.79	9.71	49.83	64.18	−0.598	Nucleus
LUSG00004384	291	31,108.22	8.94	59.32	64.81	−0.360	Nucleus
LUSG00005032	378	40,485.94	9.32	66.85	62.51	−0.406	Nucleus
LUSG00004390	239	24,894.83	5.68	66.98	65.1	−0.483	Nucleus
LUSG00005582	390	41,527.5	9.94	59.21	70.62	−0.399	Nucleus
LUSG00010997	215	22,528.05	9.77	53.72	65.53	−0.615	Nucleus
LUSG00012543	352	38,712.31	8.62	61.95	63.27	−0.786	Nucleus
LUSG00029783	285	30,498.46	9.02	51.83	64.77	−0.379	Nucleus
LUSG00029029	808	88,760.33	7.33	66.42	69.05	−0.500	Nucleus
LUSG00007909	335	36,687.08	8.74	63.12	63.28	−0.765	Nucleus

## Data Availability

The RNA-seq raw sequencing data generated in this study have been deposited in the Sequence Read Archive (SRA) of the National Center for Biotechnology Information (NCBI) under the accession number (PRJNA1442308). These data can be accessed publicly via the NCBI SRA portal (https://www.ncbi.nlm.nih.gov/sra, accessed on 3 April 2026). All other relevant data supporting the findings of this study are included in the main manuscript and its Supplementary Materials.

## Supplementary Materials

The following supporting information can be downloaded at: https://www.mdpi.com/article/10.3390/ijms27083594/s1.
