# Supplementary material for: Identification, Bioinformatics, and Expression Analysis of JAZ Gene Family in Flax (Linum usitatissimum L.)
Source: Int J Mol Sci. 2026 Apr 17;27(8):3594. doi: 10.3390/ijms27083594 (PMC13116951; doi:10.3390/ijms27083594)
Supplement: Supplementary file 1 [file ijms-27-03594-s001.zip › ijms-4110472-supplementary.pdf]

Figure S1. The homology analysis of LuJAZs.

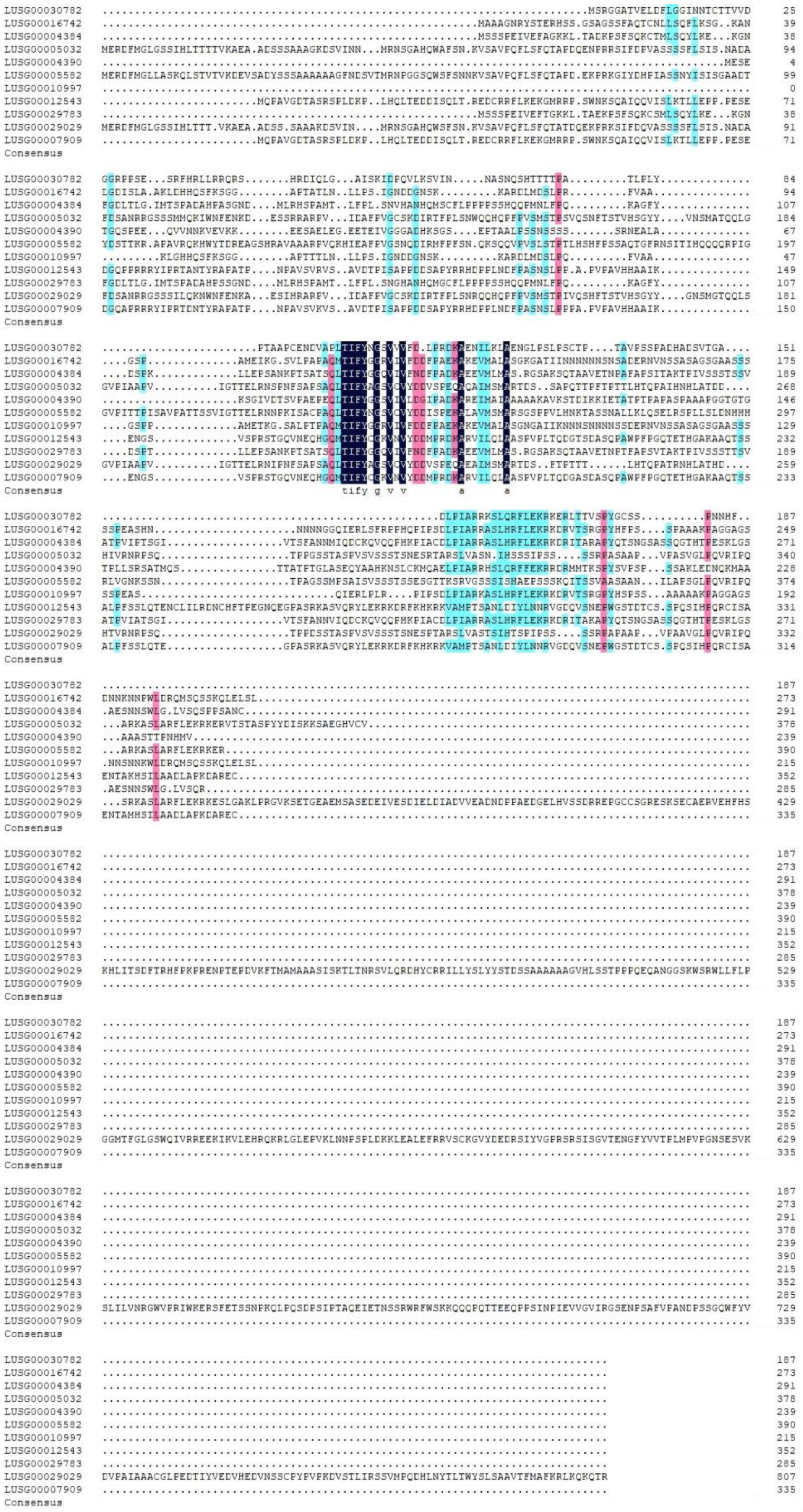

Table S1. The quality of sample sequencing data

sample	raw_reads	raw_bases（G）	clean_reads	clean_bases（G）	total_map	Q20	Q30	C_pct
C1	51104954	7.67	49933308	7.49	47514191(95.16%)	99.45	97.51	48.32
C2	50471262	7.57	49471254	7.42	47882540(96.79%)	99.41	97.37	49.25
C3	49722500	7.46	48878648	7.33	47645288(97.48%)	99.46	97.5	49.03
T12_1	49412998	7.41	48575916	7.29	47328006(97.43%)	99.42	97.45	48.35
T12_2	48287958	7.24	47493222	7.12	46332730(97.56%)	99.46	97.5	48.72
T12_3	48002732	7.2	45304084	6.8	44203245(97.57%)	99.41	97.4	48.43
T24_1	46388528	6.96	45498128	6.82	44212845(97.18%)	99.46	97.52	48.04
T24_2	51513368	7.73	50623566	7.59	49290084(97.37%)	99.49	97.63	48.39
T24_3	47530002	7.13	44903014	6.74	43801404(97.55%)	99.49	97.6	48.62
average value	49159367	7.37	47853460	7.18	46467814.78(97.12%)	99.45	97.50	48.57

Note: Q20: Percentage of bases with Phred value greater than 20 of the total bases, raw_reads: Number of reads the original data, clean_reads: Number of reads after filtering the original data, Q30: Percentage of bases with Phred value greater than 30 of total bases, total_map: Number of reads mapped to the genome and its percentage.

Figure S2. Analysis of sample repeatability; analysis of sample repeatability.

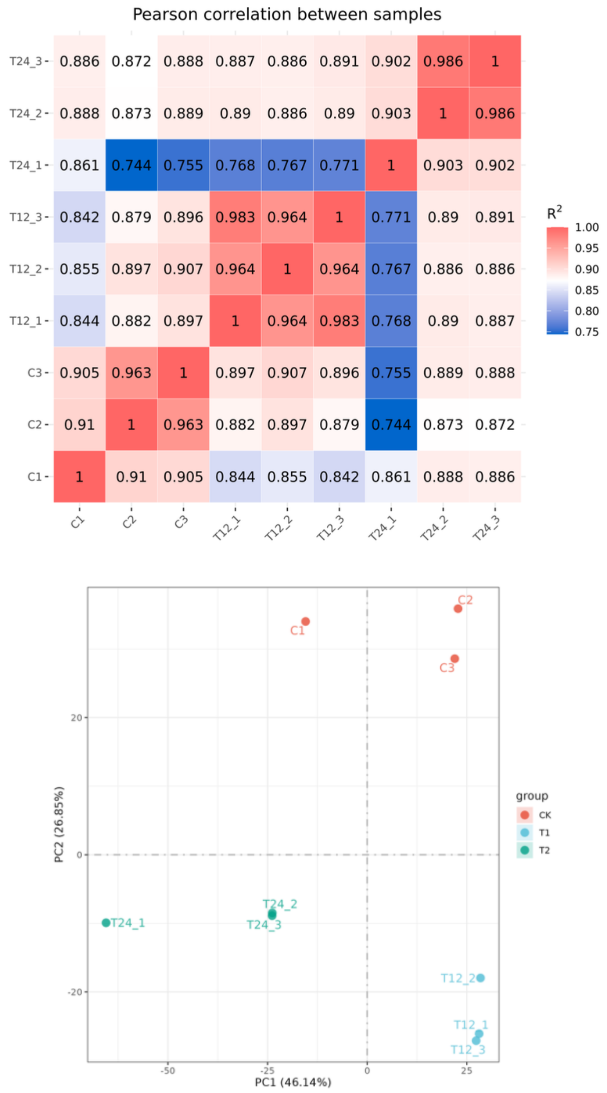

Note: (A) Heat map of inter-sample correlation; (B) Score plot of principal component analysis of the samples.

Table S2. Expression of LuJAZs genes in the transcriptome responding to MeJA.

gene_id	C1	C2	C3	T12_1	T12_2	T12_3	T24_1	T24_2	T24_3	Gene_description	Pathway (signal transduction)
LUSG00030782	1.70	2.25	2.44	20.81	7.35	22.00	4.83	5.97	6.63	TIF9_ARATH Protein TIFY 9	Plant hormone
LUSG00016742	15.93	3.55	6.53	99.51	16.51	95.14	61.75	20.55	22.10	JAZ1_SOYBN Jasmonate ZIM domain-containing protein 1	Plant hormone
LUSG00004384	12.77	6.97	6.14	27.21	12.55	29.76	29.11	19.85	19.39	TI10A_ARATH Protein TIFY 10A	Plant hormone
LUSG00005032	13.62	15.59	13.03	20.98	15.70	19.77	14.60	11.29	12.88	TIF6B_ARATH Protein TIFY 6B	Plant hormone
LUSG00004390	67.89	37.58	45.70	110.69	79.44	116.18	141.63	107.12	103.51	TIF3B_ARATH Protein TIFY 3B	Plant hormone
LUSG00005582	41.72	44.95	40.38	90.60	35.86	89.30	35.10	23.45	28.59	TIF6B_ARATH Protein TIFY 6B	Plant hormone
LUSG00010997	15.66	7.19	10.31	142.53	25.67	150.35	46.94	37.68	36.86	TI10A_ORYSJ Protein TIFY 10A	Plant hormone
LUSG00012543	5.11	4.58	3.90	4.72	4.32	5.87	4.91	4.23	4.40	TIF4A_ARATH Protein TIFY 4A	--
LUSG00029783	6.65	2.97	4.33	137.15	21.68	147.97	28.51	17.20	16.10	TI10A_ARATH Protein TIFY 10A	Plant hormone
LUSG00029029	9.18	12.58	10.19	16.60	13.74	16.20	10.77	9.37	10.48	SURF1_ARATH Surfeit locus protein 1	--
LUSG00007909	5.96	5.40	5.05	6.07	6.10	5.89	4.42	5.62	5.97	TIF4A_ARATH Protein TIFY 4A	--

Table S3. The MYC2 gene in DEGs

Time after MeJA treatment	gene_id	log2FoldChange	pvalue	gene_description	Family
12 h	LUSG00028070	2.26515781125548	6.72192028413948e-07	- && sp|A0A3Q7HRZ6|MYC2_SOLLC Transcription factor MYC2 OS=Solanum lycopersicum OX=4081 GN=MYC2 PE=1 SV=1 && PF00010:Helix-loop-helix DNA-binding domain|PF14215:bHLH-MYC and R2R3-MYB transcription factors N-terminal	bHLH
	LUSG00000501	1.8041250883231	7.86326342911799e-07	- && sp|K4PW38|RSS3_ORYSJ Protein RICE SALT SENSITIVE 3 OS=Oryza sativa subsp. japonica OX=39947 GN=RSS3 PE=1 SV=1 && PF14215:bHLH-MYC and R2R3-MYB transcription factors N-terminal	bHLH
24 h	LUSG00033635	4.07676670103524	6.92506523301503e-11	- && sp|Q9CAD0|EGL1_ARATH Transcription factor EGL1 OS=Arabidopsis thaliana OX=3702 GN=BHLH2 PE=1 SV=1 && PF14215:bHLH-MYC and R2R3-MYB transcription factors N-terminal	bHLH
	LUSG00028070	1.88239306292285	6.33293348540001e-05	- && sp|A0A3Q7HRZ6|MYC2_SOLLC Transcription factor MYC2 OS=Solanum lycopersicum OX=4081 GN=MYC2 PE=1 SV=1 && PF00010:Helix-loop-helix DNA-binding domain|PF14215:bHLH-MYC and R2R3-MYB transcription factors N-terminal	bHLH

Table S4. The primer sequences of qRT-PCR

Primers	Sequence	Tm	Amplicon
LUSG00030782-F	TCAGAGAAGCCACCGAGATA	62.05	94
LUSG00030782-R	CGACTGGTTGGAGGCATTAT	61.98	
LUSG00016742-F	GGAGAAAGCGAAGGAGGTTATG	62.50	97
LUSG00016742-R	GTTACGCTCATCAGCCGAATTA	62.64	
LUSG00004384-F	CCAGCCTCTGGGAATGATATG	61.99	111
LUSG00004384-R	AGAAGGAGGTGGAGGAAGAA	62.09	
LUSG00004390-F	GAGGGTGAAGAGACTGAGATTG	61.69	97
LUSG00004390-R	AGGAGGAGGAGGAGTTAGAAG	61.67	
LUSG00010997-F	GACGACGGAAACTCGAAGAA	61.83	107
LUSG00010997-R	GTAGGAAGAGCAGAGCCTTTAG	61.74	
LUSG00029783-F	TGATAAGGCGGAGGAAGTTATG	61.77	96
LUSG00029783-R	ACTGATGGTGCAAAGGTAGG	61.92	
LuActin-F	GGTGTTATGGTTGGAATGGGTC	64.30	200
LuActin-R	CCTCAGTGAGAAGTACAGGGTG	64.80	
